# P3-MSDA: Multi-Source Domain Adaptation Network for Dynamic Visual Target Detection

**DOI:** 10.3389/fnhum.2021.685173

**Published:** 2021-08-09

**Authors:** Xiyu Song, Ying Zeng, Li Tong, Jun Shu, Guangcheng Bao, Bin Yan

**Affiliations:** ^1^Henan Key Laboratory of Imaging and Intelligent Processing, Chinese People's Liberation Army (PLA) Strategic Support Force Information Engineering University, Zhengzhou, China; ^2^Key Laboratory for NeuroInformation of Ministry of Education, School of Life Science and Technology, University of Electronic Science and Technology of China, Chengdu, China

**Keywords:** brain-computer interface, P3 detection, individual transfer, domain adaptation, EEG

## Abstract

Single-trial electroencephalogram detection has been widely applied in brain-computer interface (BCI) systems. Moreover, an individual generalized model is significant for applying the dynamic visual target detection BCI system in real life because of the time jitter of the detection latency, the dynamics and complexity of visual background. Hence, we developed an unsupervised multi-source domain adaptation network (P3-MSDA) for dynamic visual target detection. In this network, a P3 map-clustering method was proposed for source domain selection. The adversarial domain adaptation was conducted for domain alignment to eliminate individual differences, and prediction probabilities were ranked and returned to guide the input of target samples for imbalanced data classification. The results showed that individuals with a strong P3 map selected by the proposed P3 map-clustering method perform best on the source domain. Compared with existing schemes, the proposed P3-MSDA network achieved the highest classification accuracy and F1 score using five labeled individuals with a strong P3 map as the source domain. These findings can have a significant meaning in building an individual generalized model for dynamic visual target detection.

## Introduction

Brain-computer interface has developed a new way for human beings to communicate and control the outside world, and has a great application value and development potential in the fields of medical rehabilitation (Pan et al., 2018), recreation (Polina et al., 2018), and public safety (Ward and Obeid, 2018). Electroencephalogram (EEG) has become one of the most popular neuroimaging technologies for brain-computer interface (BCI) application because of its high temporal resolution, low cost, and portability. Influenced by external environment changes and the state of the human body, EEG signals are usually non-stationary, which increases the difficulty of single-trial EEG analysis. Besides, data distributions vary across individuals, restricting the generalization of computing models (Kaur et al., 2019; Lorena et al., 2019). It can be further aggravated for single-trial EEG detection in dynamic video target detection because of the absence of an explicit target onset time, time jitter of the detection latency, dynamics of visual background, and uncertainty of visual distracters (Song et al., 2020). In addition, more ERP components induced by dynamic visual targets contain P1, P2, P3, and a strong negative wave at around 500 ms. All of these could further influence detection performance and enlarge individual difference for dynamic visual target detection. Thus, it is essential to investigate the individual transfer problem, to build an individual generalized model, which will make the video target detection BCI system more applicable in real life.

Traditional machine learning often requires sufficient labeled training samples satisfying independent and identically distributed conditions with test samples to ensure classifier reliability, which increases the preparation time for a specific test individual, causing time consumption, and results in other individual labeled data going to waste. However, transfer learning can provide an effective way to overcome these problems (Pan and Qiang, 2010). In transfer learning, the labeled and unlabeled samples are regarded as source and target domains, respectively. By mapping the source and target domains to the same distribution space, transfer learning can reduce the domain shift between the source and target domains and use the knowledge learned from the source domain to solve different but related target domain classification problems, indicating a way to solve individual differences in EEG signals (Chen et al., 2020; Gao et al., 2020; D et al., 2021). Studies on individual difference in EEG signals based on transfer learning can be described as the problem of EEG-based individual transfer. The transfer problem with consistent feature and category spaces but inconsistent distribution space can be solved by domain adaptation in transfer learning. The core idea of domain adaptation is to eliminate cross-domain distribution differences. Domain adaptation relaxes the condition that training samples and test samples are independent and identically distributed in traditional machine learning. Knowledge is transferred from the labeled source domain to the unlabeled target domain to reduce the annotation cost, a typical unsupervised domain adaptation problem. The unsupervised domain adaptation method has a rich research foundation in the field of computer vision (Pan et al., 2011; Gong et al., 2015; Csurka, 2017). Early unsupervised domain adaptation criteria assume that all source domain data arise from the same source with the same distribution. It is easier to collect labeled data from multiple source domains with different distributions. The data from multiple source domains can transfer more information to the target domain, which will be more beneficial in practical applications (Jhuo et al., 2013; Ackaouy et al., 2020). Thus, this study adopts multi-source domain adaptation (MSDA) to solve the individual transfer on the task of dynamic visual target detection.

With the rapid development of convolutional neural network (CNN), MSDA has been widely investigated because of its practice feasibility and performance superiority. The ability of category recognition and domain transformation should be considered in MSDA networks, which bring challenges to the design. Based on the existing domain adaptation network (Ganin et al., 2017), scholars proposed a series of MSDA criteria, which shared feature extractors for different source domains. Zhao et al. proposed multi-source domain adversarial networks where each source domain feature from the common feature extractor was aligned with the target domain feature, and a category classifier was trained using the features of all source domains (Zhao et al., 2018). Xu et al. proposed a deep cocktail network, which included multi-source domain discriminators to narrow the gap between each source and target domain and multi-source category classifiers to predict categories from different source domains. The confusion score was calculated based on the loss of each discriminator to calculate the weight of each classifier (Xu et al., 2018). Peng et al. proposed moment matching for MSDA (M3SDA), which introduced a moment matching principle between source domains to better eliminate the domain differences between multi-source domain and target domain, and used the classification accuracy of each source domain to calculate the classifier weight for target domain prediction (Peng et al., 2019). Li et al. integrated the existing multi-source domain adaptive network strategy and proposed mutual learning network for multi-source domain adaptation (ML-MSDA) network, including multiple branch networks and a guidance network, where the single source and target domains, and combined source and target domains were aligned by conditional adversarial adaptation. The unsupervised classification loss of the target domain was introduced. Thus, cross-domain information adaptability and network robustness were enhanced to achieve the target domain classification with the guidance network as the center (Li et al., 2020b). These studies provide valuable guidance for designing transfer networks in BCI systems.

Because of remarkable achievements of the domain adaptation method in computer vision, researchers have started paying attention to its application in the field of BCI (Wang et al., 2015; Wu, 2017; Wu et al., 2020; Cao et al., 2021). For EEG-based domain adaption, current studies focus on emotion recognition, cognitive load recognition, movement recognition, and motor imagery decoding. Li et al. and Bao et al. investigated multi-source transfer learning and two-level domain adaptation neural networks, respectively, for cross-subject EEG emotion recognition (Li et al., 2019; Bao et al., 2021). Jimenez-Guarneros et al. proposed custom domain adaptation for cross-subject cognitive load recognition (Jimenez-Guarneros and Gomez-Gil, 2020). McKendrick et al. focused exclusively on labeling cognitive load data for supervised three-state classification (McKendrick et al., 2019). Li et al. proposed a principal component analysis (PCA)-based MSDA (PMDA) algorithm for cross-time movement classification (Li et al., 2020a). Tang et al. proposed a novel conditional domain adaptation neural network framework for motor imagery EEG signal decoding. A densely connected CNN is used to obtain high-level discriminative features from raw EEG time series (Tang and Zhang, 2020). However, there is little research on the task of EEG-based target detection, which might be caused by the difficulty of training on an imbalanced dataset of EEG-based target detection. The existing domain adaptation networks mainly consider the classification problem of balanced samples; however, the samples of EEG-based target detection are inherently imbalanced, where the target is a low-probability event. Besides, data from different individuals as source domains may influence classification performance. In the existing studies, there is lack of reasonable criteria for individual selection of source domain. Thus, it is necessary to design an individual generalized MSDA network with a certain criterion for source domain selection to achieve dynamic visual target detection.

In this study, we proposed an unsupervised multi-source domain adaptation network (P3-MSDA) to develop an individual generalized model for dynamic visual target detection. We designed a P3 map-clustering method to select individuals with a strong P3 map as the source domain. Domain shift was eliminated using the condition domain confrontation networks. The multi-source domain classifiers were weighted to predict target domain categories. Furthermore, the ensemble prediction probabilities were ranked and returned to guide the input of input of target samples to solve the problem of imbalanced data classification.

## Experiment and Recordings

### Experimental Paradigm of UAV Video-Vehicle Detection

Thirty-four healthy college participants were recruited for this experiment with an average age of 25 years. All the participants signed the informed consent before the experiment. The experiment was approved by the ethics committee of Henan Provincial People's Hospital.

**Figure 1:**
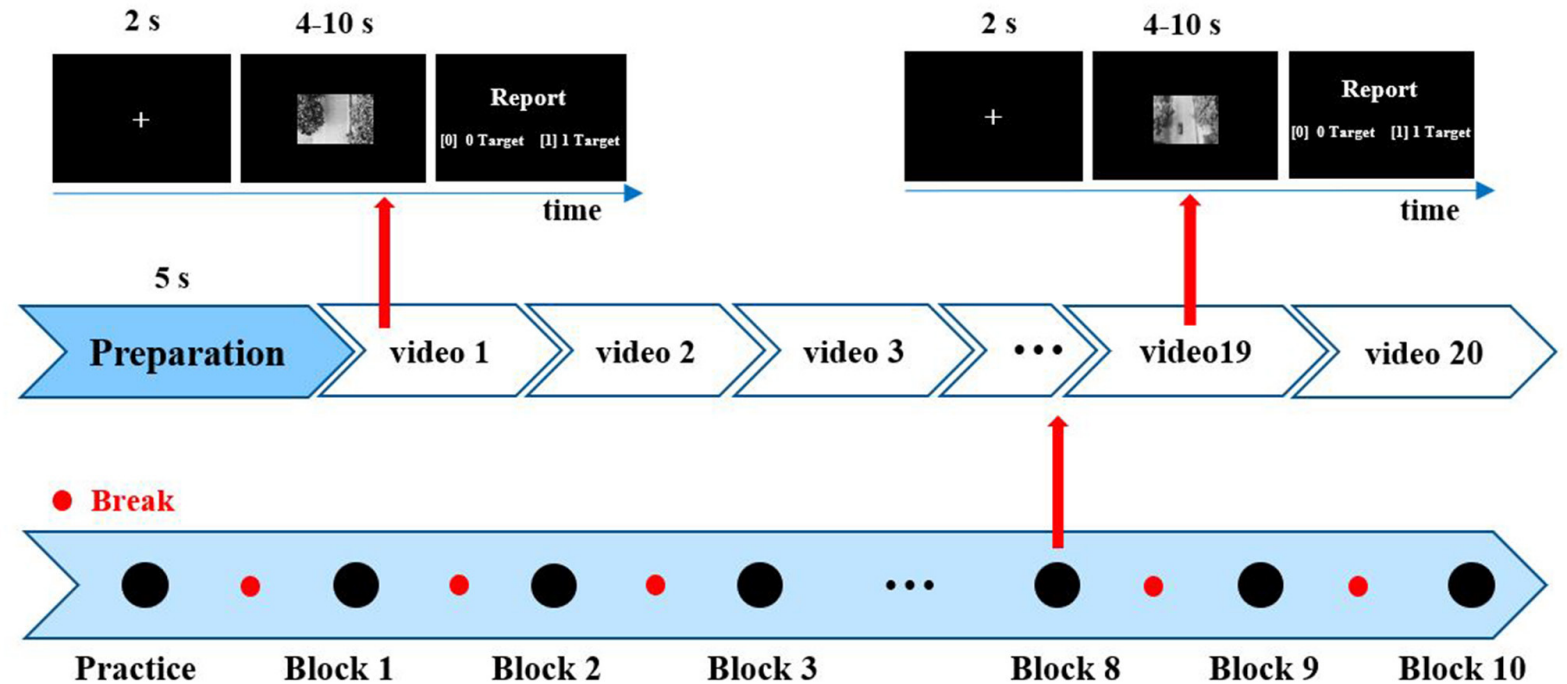
Paradigm of unmanned aerial vehicle (UAV) video-vehicle detection. Two hundred video clips were divided into 10 blocks, including 10 deviant videos and 10 standard videos per block. After each clip, participants reported vehicle numbers by the keypress. “1” denotes one vehicle, and “0” denotes no vehicle.

EEG data were induced by unmanned aerial vehicle (UAV) video in the task of video-vehicle detection. The paradigm of UAV video-vehicle detection is shown in Figure 1 and described in previous studies (Song et al., 2020). The UAV flew along a wide street and recorded. The experiment consists of 200 video clips where 100 video clips contain one vehicle as the deviant videos, and the remaining 100 clips without a vehicle are regarded as standard videos. Besides, 200 video clips were uniformly arranged into 10 blocks where deviant and standard video clips were randomly presented. The duration of each video was 4–10 s. In the experiment, the participants were required to quickly find vehicles regardless of other distractors, and report vehicle numbers for each clip. In future study, we will share the dataset with peers.

### Data Preprocessing

An EEG recording system (g.HIamp, g.tec medical engineering Gmb H, Schiedlberg, Austria) was used to record EEG data using 64 Ag/AgCl electrodes according to the extended 10–20 system with 61 valid channels. The online sample rate was 600 Hz with a band-pass filter of 0.01–100 Hz and a notching filter of 50 Hz. Signals were filtered to 0.1−30 Hz and resampled to 100 Hz after ICA-based artifact removal. The average reference was used. EEG signals for false reported videos were removed. To ensure the reliability of sample labels, we intercepted deviant samples from deviant video-induced EEG signals and standard samples from standard video-induced EEG signals. Deviant samples were intercepted for 1,500 ms, starting from the target onset time. Since there was only one vehicle in each deviant video, one deviant sample could be obtained from one deviant video. Standard samples were intercepted from standard video-induced signals without overlapping. In this way, several standard samples can be obtained from one standard video. With an ERP alignment method proposed in the previous studies (Song et al., 2020), these samples were aligned and intercepted to 1,000 ms single-trial signals whose amplitudes over ± 100 μV were discarded. After these, there were around 300–500 valid trials for each participant. The ratio of the deviant trials to standard trials is 1: 4.1–4.5.

## P3-MSDA Network

**Figure 2:**
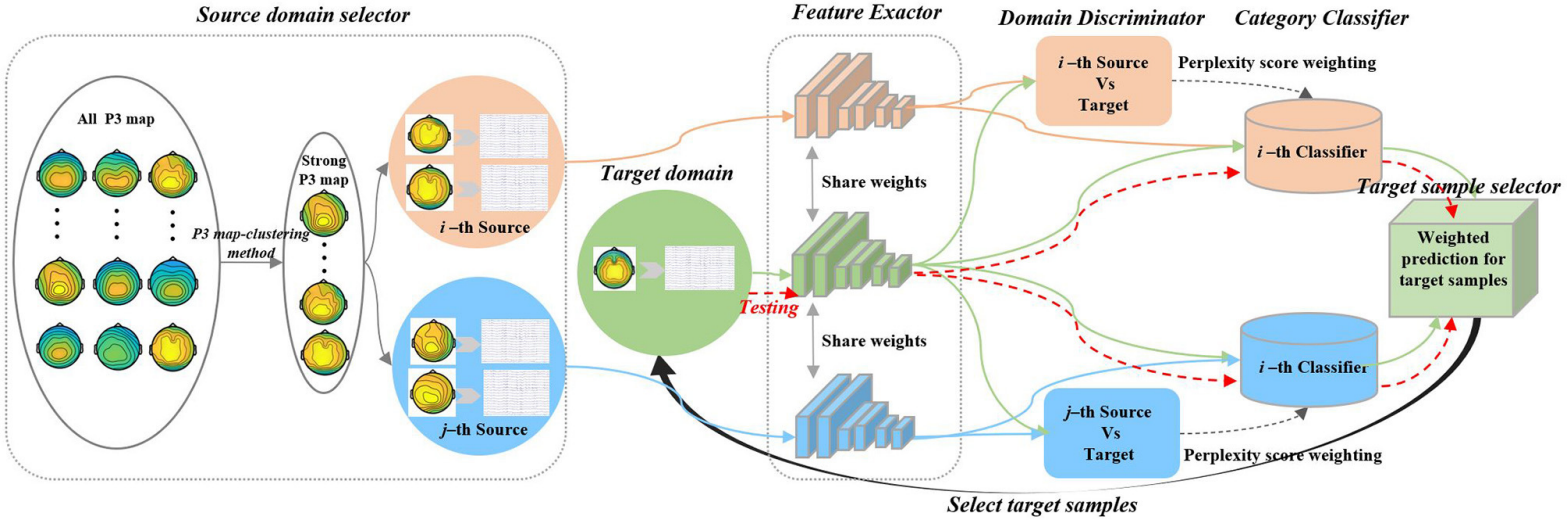
P3-MSDA network architecture. The framework selects individuals to construct the multi-source domains based on the P3 map-clustering method and adapts the target domain to these multi-source domains to achieve individual transfer for dynamic visual target detection. For simplicity, we consider the *i*-th and *j*-th sources. The feature extractor maps target and source samples into a common feature space. After a gradient reversal processing, the *i*-th domain discriminator offers the *i*-th adversary between the *i*-th source and target features. Similarly, the *j*-th domain discriminator offers the *j*-th adversary between the *j*-th source and target features. The *i*-th category classifier produces the category results for the *i*-th sources and target domain. The target classification operator integrates all weighted classification results and predicts the target category where the weight is calculated based on the domain shift. The prediction probability can further guide the input of target samples for the next iteration.

This study predicts the category of samples in the target domain using the data distribution and labels of the source domain and the data distribution of the target domain. Aiming at an individual generalized model for dynamic visual target detection, the historical data from existing individuals with the labeled dataset are used to construct source domains. Besides, the new individual with an unlabeled dataset is regarded as the target domain. With a domain adaption network, we can directly predict the labels of the new individual using historical data to reduce the calibration time. In this study, we propose a P3-MSDA network, where a P3 map-clustering method is presented to select individuals as the source domain. Adversarial domain adaptation is conducted for domain alignment to eliminate individual differences. The prediction probability is ranked and returned to guide the input of target samples for the imbalanced data classification. The framework consists of five parts: source domain selector, feature extractor, domain discriminator, category classifier, and target sample selector, as shown in Figure 2.

There are *N* source domains and one target domain where the target domain covers most part of the testing data. For the *j*-th source domain, the data distribution submits to *p*_*s*_*j*__(**X**_*s*_*j*__, **Y**_*s*_*j*__). The labeled samples are $\left({\text{X}}_{s_{j}},{\text{Y}}_{s_{j}}\right)\text{=}{\left\{\left(x_{k}^{s_{j}},y_{k}^{s_{j}}\right)\right\}}_{k=1}^{n_{s_{j}}}$ where **X**_*s*_*j*__ and **Y**_*s*_*j*__ denote the sample set and the corresponding label vector, respectively. $x_{k}^{s_{j}}$denotes the single-trial EEG matrix. *n*_*s*_*j*__ is the sample number for the *j*-th source domain. $y_{k}^{s_{j}}$ denotes the category label of two categories for the *k*-th sample. The samples and labels of the target domain are (**X**_*t*_, **Y**_*t*_) with data distribution *p*_*t*_ (**X**_*t*_, **Y**_*t*_). The data distribution between the source and target domain is similar but also different. The similarity ensures that they can share the feature extraction for classification, and the difference is usually expressed as domain shift (Adams, 2010), which will be eliminated by domain adaption network.

### Source Domain Selector

The source domain selector selects individuals as source domains for robust network training. The brain map at the P3 peak time can reflect the brain activities of decision-making, since the P3 component is regarded as the key feature for EEG-based target detection. Thus, a P3 map-clustering method is proposed for source domain selection.

Since we dealt with EEG signals induced by video clips with random vehicles, P3 latency may vary a lot among different trials. Therefore, an ERP alignment method was used to eliminate latency jitter before individual clustering because of the severe latency jitter of video-induced P3 signals (Song et al., 2020).

The individual ERP was calculated by averaging aligned deviant trials. Then, the brain map at the P3 peak time was extracted as a feature vector with the same size as the channel number. Based on a distance-clustering method, all individuals can be clustered into three groups: strong, medium, and weak P3 map groups. Here, individuals with a strong P3 map were selected to construct the source domain.

### Feature Extractor

**Figure 3:**
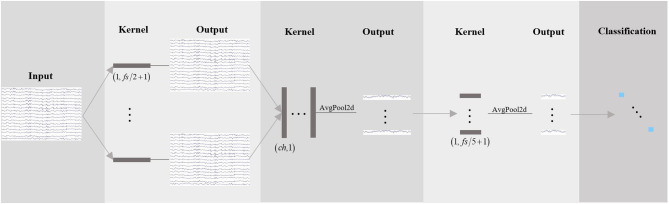
Feature extractor network. There are three convolution operations. The network starts with temporal convolution (the first convolution operation) to learn frequency filters, then uses a spatial convolution (the second convolution operation) to learn spatial filters. The third convolution learns a temporal summary for all feature maps.

The feature extractor maps all samples from the target domain and *N* source domains into a common feature space to obtain the best mapping between all source domains and target domain, thus, successfully learning the domain invariant features. The network structure is shown in Figure 3. The input samples $x_{k}^{s_{i}}$, $x_{k}^{s_{j}}$, and $x_{k}^{t}$ are a matrix with a size of *c* × *T*. *c* is the channel number, and *T* is the number of time points with *fs* sample rate. The feature is extracted with three convolution operations. The first convolution kernel size is 1 × (*fs*/2 + 1) for band-pass filters with around 2 Hz frequency resolution. The size of the second convolution kernel is *c* × 1 for reducing the spatial dimension from multiple channel signals to time-series. After the average pooling layer with double downsampling, the size of the third convolution is 1 × (*fs*/5 + 1), and the sliding sum of 200 m sec data in time and further down sampling to *fs*/10 Hz. The obtained feature is transformed into a one-dimensional feature vector expressed as $F\left(x_{k}^{s_{i}}\right)$ for the *i*-th source domain or $F\left(x_{k}^{t}\right)$ the target domain. Details of the feature extractor architecture are presented in Table 1.

**Table 1:** Network parameters of feature extractor.

**Layer**	**Filter**	**Size**	**Output**	**Option**
Input	–	–	(1, *ch, T*)	–
Conv2d	*F*_1_	(1, *fs*/2 + 1)	(*F*_1_, *ch, T*)	–
BatchNorm2d	–	–	(*F*_1_, *ch, T*)	–
Conv2d	*F*_2_	(*ch*, 1)	(*F*_2_, 1, *T*)	–
BatchNorm2d	–	–	(*F*_2_, 1, *T*)	–
ReLU	–	–	(*F*_2_, 1, *T*)	–
AvgPool2d	–	(1, 2)	(*F*_2_, 1, *T*/2)	–
Conv2d	*F*_2_	(1, *fs*/5 + 1)	(*F*_2_, 1, *T*/2)	–
BatchNorm2d	–	–	(*F*_2_, 1, *T*/2)	–
ReLU	–	–	(*F*_2_, 1, *T*/2)	–
AvgPool2d	–	(1, 5)	(*F*_2_, 1, *T*/10)	–
Dropout2d	–	–	(*F*_2_, 1, *T*/10)	*p*_*dropout*_
Flatten	–	–	(*F*_2_ × 1 × *T*/10)	-

### Domain Discriminator

The domain discriminator distinguishes the distribution distance between the target domain and the source domain by adversary learning. In this study, to eliminate domain shift, a gradient reversal layer (Ganin et al., 2017) is introduced. For the convenience of domain adaptation neural network, it is a positive function in forward computing, while it is a negative function in backpropagation. For *N* source domains, there will be *N* domain discriminators ${\left\{D^{s_{j},t}\right\}}_{j=1}^{N}$ to map all source domains and target domain to a common feature space. The features $F\left(x_{k}^{t}\right)$ and $F\left(x_{k}^{s_{j}}\right)$ from feature extractors were discriminated by the domain discriminator $D^{s_{j},t}$ to identify whether they come from the distribution of the same domain. The domain discriminators $D^{s_{j},t}$ adopt a full connected layer, and the number of neurons is *F*_2_ × 1 × *T*/10. The discriminant probabilities of the target samples and source samples are $p_{k}^{D^{s_{j},t},t}\text{=}D^{s_{j},t}\left(F\left(x_{k}^{t}\right)\right)$$p_{k}^{D^{s_{j},t},s_{j}}\text{=}D^{s_{j},t}\left(F\left(x_{k}^{s_{j}}\right)\right)$. Using cross-entropy loss with a softmax activation function, the domain discrimination loss between the target domain and*j*-thsource domain is given as follows:

(1) $$\begin{array}{r} L_{adv}^{s_{j},t}=-\overset{K}{\underset{k=1}{\sum }}\left(v_{s}\log p_{k}^{D^{s_{j},t},s_{j}}\text{+}v_{t}\log p_{k}^{D^{s_{j},t},t}\right) \end{array}$$

where *v*_*s*_ and *v*_*t*_ are a one-hot label for target and source domain. *K* is the batch size.

### Category Classifier

The category classifiers ${\left\{C_{j}\right\}}_{j=1}^{N}$distinguish between deviant and standard samples from target and source domains. They are shaped with a full connection layer of *F*_2_ × 1 × *T*/10 neurons. The features $F\left(x_{k}^{t}\right)$ and $F\left(x_{k}^{s_{j}}\right)$ from feature extractors were classified by the classifier *C*_*j*_, then the category predictions are denoted as $p_{k}^{C_{j},t}=C_{j}\left(F\left(x_{k}^{t}\right)\right)$ for the target domain and $p_{k}^{C_{j},s_{j}}=C_{j}\left(F\left(x_{k}^{s_{j}}\right)\right)$ for the *j*-th source domain. For the labeled source domains, we can use supervised cross-entropy loss to perform training. The category loss of the source domain is given as follows:

(2) $$\begin{array}{r} L_{class}^{C_{j},s_{j}}=-\overset{K}{\underset{k=1}{\sum }}\left(y_{k}^{s_{j}}\log p_{k}^{C_{j},s_{j}}\right) \end{array}$$

where $y_{k}^{s_{j}}$ is the category label of the*k*-thsample from the*j*-thsource domain.

For the unlabeled target domain, we use unsupervised entropy loss to include them in the classifier training. The category loss of the target domain is given as follows:

(3) $$\begin{array}{r} L_{class}^{C_{j},t}=-\overset{K}{\underset{k=1}{\sum }}\left({ŷ}_{k}^{t}\log p_{k}^{C_{j},t}\right) \end{array}$$

where ${ŷ}_{k}^{t}$ is the predicted pseudo label of the *k*-th sample from the target domain, which is binarized from the prediction probability ${\hat{p}}_{k}^{t}$, an ensemble prediction probability from the target classification operator.

### Target Sample Selector for Imbalanced Data Classification

Because of the inherent sample imbalance in EEG-based target detection, we have to adjust the sample selection strategy of DA networks to prevent the model from bias to the standard samples. In this study, all deviant and randomly equivalent standard samples from source domains can be selected for sample balance in each epoch, since the label vector of the source domain is available. There is only a prediction probability for the target domain. Based on prediction probability, all the testing data are ranked from high to low according to the probability value predicted as deviant stimuli. The first *Q* samples from the testing data are used as target domain samples in the next iteration, which can dynamically employ the target samples as much as possible and overcome model bias to standard samples to some extent.

For the target sample $x_{k}^{t}$, *N* category classifiers can give category predictions $\left\{p_{k}^{{}^{C_{1},t}},\ldots ,p_{k}^{{}^{C_{j},t}},\ldots ,p_{k}^{{}^{C_{N},t}}\right\}$, which will be weighted for a prediction probability ${\hat{p}}_{k}^{t}$ based on the perplexity score of domain discriminators. From the literature (Xu et al., 2018), the target-source perplexity score of the *j*-th source domain is calculated as follows:

(4) $$\begin{array}{r} ps_{j}\text{=}-\text{log}\left(1-D^{s_{j},t}\left(F\left(x_{k}^{t}\right)\right)\right)\text{+}l_{s_{j}} \end{array}$$

where *l*_*s*_*j*__ is obtained by averaging the discriminator loss of the *j*-th source domain. The classifier weight is the normalization value of the perplexity score:

(5) $$\begin{array}{r} w_{j}=ps_{j}\;/\overset{N}{\underset{j=1}{\sum }}ps_{j}\; \end{array}$$

Thus, the prediction probability of the *k*-th target sample is given as follows:

(6) $$\begin{array}{r} {\hat{p}}_{k}^{t}=\frac{1}{N}\overset{N}{\underset{j-1}{\sum }}\left(w_{j}\times p_{k}^{{}^{C_{j},t}}\right) \end{array}$$

which is also binarized as prediction category and training pseudo labels ${\hat{\text{Y}}}_{t}$.

By integrating the domain discriminator and category prediction losses, we obtain the following adversarial learning problem:

(7) $$\begin{array}{r} \underset{F,\;D,\;C}{\min}\;\alpha \overset{N}{\underset{j=1}{\sum }}L_{adv}^{s_{j},t}+\gamma \overset{N}{\underset{j=1}{\sum }}L_{class}^{C_{j},s_{j}}+\lambda \overset{N}{\underset{j=1}{\sum }}L_{class}^{C_{j},t} \end{array}$$

where α, γ, and λ are hyper-parameters. $L_{adv}^{s_{j},t}$, $L_{class}^{C_{j},s_{j}}$, and $L_{class}^{C_{j},t}$ denote domain loss, category loss on source domains, and category loss on the target domain, respectively.

### The Training and Testing Process of the Network

The pseudocode of the P3-MSDA algorithm is presented in Table 2. In the training stage, source domain individuals are selected to align with the target domain for target sample classification. The ensemble prediction probability from *N* category classifiers can guide the input of target samples. The network parameters are updated using the loss in Eq (7). In the testing stage, all the testing data are used to predict detection performance, as the red line shows in Figure 2.

**Table 2:** Pseudocode of theP3-MSDA algorithm.

**Learning algorithm for P3-MSDA**
**Input:** The labelled samples (**X**_0_, **Y**_0_)from all available source domain individuals and the unlabelled samples **X**_*t*_ from target domain individuals. **Output:** Well-trained feature extractor *F*, domain discriminator${\left\{D^{s_{j},t}\right\}}_{j=1}^{N}$, category classifier${\left\{C_{j}\right\}}_{j=1}^{N}$, and prediction label ${\hat{\text{Y}}}_{t}$ for target samples.
**Training stage:**
1: **for** 1: *t***do** *(t is epochs)*
2: Select source domain individuals (**X**_*s*_, **Y**_*s*_)using a P3 map-clustering method from (**X**_0_, **Y**_0_). Divide source domain individuals into*N*source domains as ${\left\{\left({\text{X}}_{s_{j}},{\text{Y}}_{s_{j}}\right)\right\}}_{j=1}^{N}$.
3: Select all deviant samples and randomly equivalent standard samples ${\left\{\left({{\text{X}}^{\prime }}_{s_{j}},{{\text{Y}}^{\prime }}_{s_{j}}\right)\right\}}_{j=1}^{N}$from ${\left\{\left({\text{X}}_{s_{j}},{\text{Y}}_{s_{j}}\right)\right\}}_{j=1}^{N}$ as source domain and the first *Q* samples ${{\text{X}}^{\prime }}_{t}$ from${\text{X}}_{t}^{\text{rank}}$as target domain. The initialization of ${\text{X}}_{t}^{\text{rank}}$ is **X**_*t*_.
4: **for** 1: *m***do***(m is batch times with batch size K)*
5: Sample batch ${\left\{x_{k}^{s_{i}},y_{k}^{s_{i}}\right\}}_{k=1}^{K}$ from ${\left\{\left({{\text{X}}^{\prime }}_{s_{j}},{{\text{Y}}^{\prime }}_{s_{j}}\right)\right\}}_{j=1}^{N}$and ${\left\{x_{k}^{t}\right\}}_{k=1}^{K}$ from ${{\text{X}}^{\prime }}_{t}$.
6: Compute domain loss ${\left\{L_{\text{adv}}^{s_{j},t}\right\}}_{j=1}^{N}$ by Eq(1) for each source domain and target domain.
7: Compute category loss ${\left\{L_{\text{class}}^{C_{j},s_{j}}\right\}}_{j=1}^{N}$ by Eq(2) for each source domain.
8: Compute classifier weight ${\left\{w_{j}\right\}}_{j=1}^{N}$ by Eq(5).
9: Compute prediction probability ${\left\{{\hat{p}}_{k}^{t}\right\}}_{k=1}^{K}$ by Eq(6).
10: Compute prediction loss ${\left\{L_{\text{class}}^{C_{j},t}\right\}}_{j=1}^{N}$ by Eq(3) for target domain.
11: Update *F*,${\left\{D^{s_{j},t}\right\}}_{j=1}^{N}$, and ${\left\{C_{j}\right\}}_{j=1}^{N}$ by Eq(7).
12: **end for**
13: Evaluate the target samples **X**_*t*_for prediction probability ${\hat{\text{p}}}^{t}$. According to the probability value predicted as deviant stimuli, samples from target domain are ranked from high to low as ${\text{X}}_{t}^{rank}$for next epoch. Binary prediction probability ${\hat{\text{p}}}^{t}$ as category label vector${\hat{\text{Y}}}_{t}$.
14: **end for**
**Testing stage:**
All the unlabelled samples **X**_*t*_ from target domain individuals are used as testing data. Using the well-trained feature extractor F and category classifier${\left\{C_{j}\right\}}_{j=1}^{N}$, the testing labels are predicted as ${\hat{\text{Y}}}_{t}$.

## Results

### Implementation Details

Theexperiments were implemented in the PyTorch platform. The sample rate of input signals is *fs* = 100 Hz with 1 s length, sample points *T* for single-trial signals are 100, and channel number *ch* is 61. The number of neurons in the fully connected layer for domain discriminators and category classifiers is 80. The feature extraction parameters stated in Table 1 are set as *F*_1_ = 4, *F*_2_ = 8, and *p*_*dropout*_ = 0.2. For the proposed P3-MSDA algorithm, we set the trade-off hyper-parameters (α,γ, λ) as (0.2, 0.8, 0.2), respectively. We fit the model using the Adam optimizer algorithm and run 300 training iterations (epochs). The learning rate is set to 0.0003. The batch size is *K* = 20, meaning that 20 samples from each source domain are conducted in domain alignment with 20 samples from the target domain. For the labeled source domain, there are equivalent deviant and standard samples in each epoch. For the unlabeled target domain, 20 samples are randomly selected from the first *Q* = 80% samples with the highest prediction probability as deviant stimuli.

**Figure 4:**
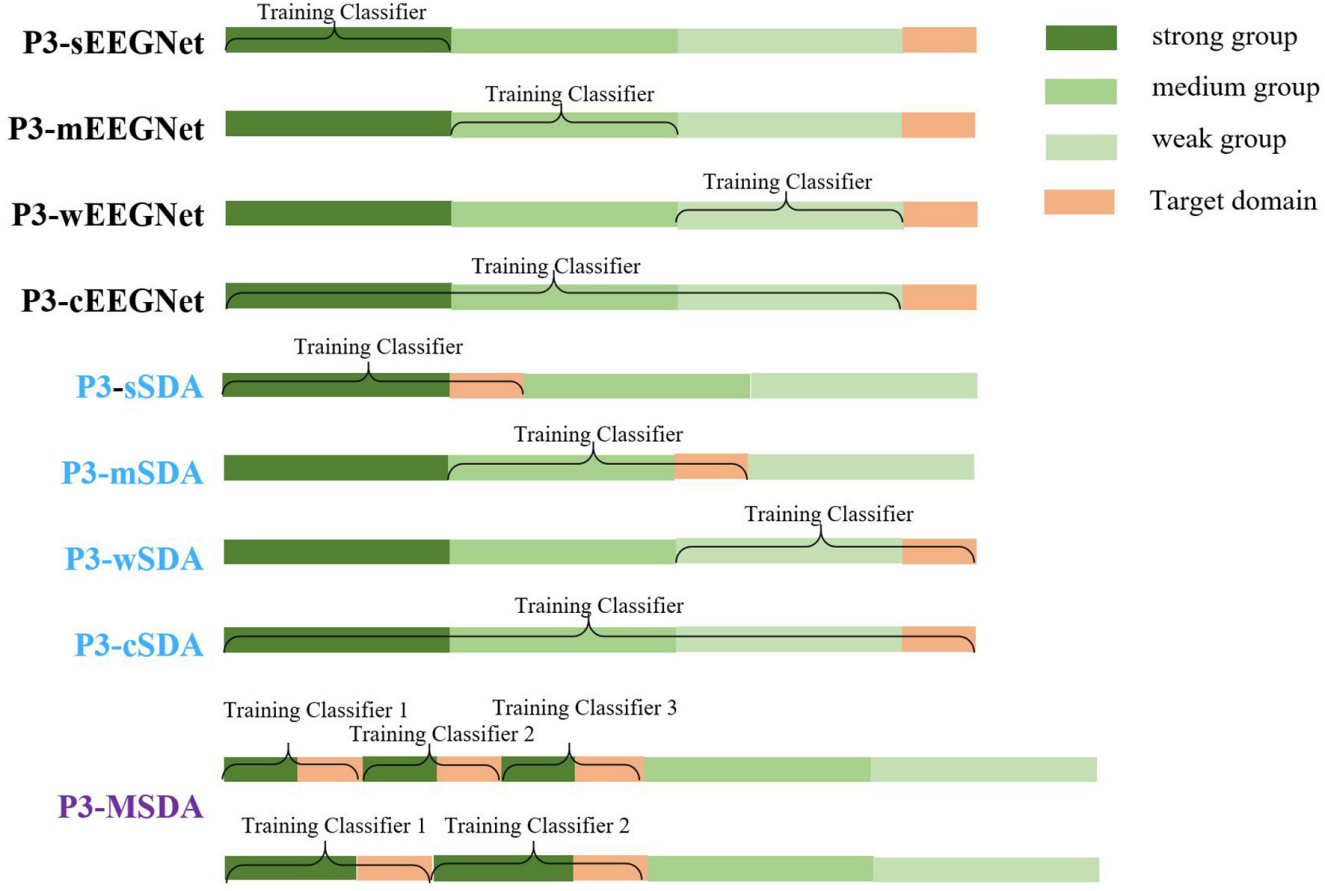
Diagram of the composition of source and target domains for all schemes. The EEGNet networks with single groups (P3-sEEGNet, P3-mEEGNet, and P3-wEEGNet) used data from the corresponding group to train the classifier without domain adaption. The single source domain adaption schemes (P3-sSDA, P3-mSDA, and P3-wSDA), respectively aligned the data from the corresponding source domain with the data from the target domain. The P3-cEEGNet scheme based on the combination of strong, medium, and weak groups used data from all groups training the classifier without domain adaption. Besides, the P3-cSDA scheme based on the combination of strong, medium, and weak groups aligned the data from the corresponding source domain with the data from the target domain. P3-MSDA based on multiple individuals or multiple sub-groups from the strong group individuals aligned the data from the multiple source domains with the data from the target domain.

To evaluate the proposed P3-MSDA, more schemes are presented and compared. To clearly describe these schemes, a diagram of the composition of the source and target domains is shown in Figure 4, where EEGNet (Lawhern et al., 2016) is ever proposed as a good generalized framework for P3 detection in online testing. However, the SDA and MSDA are offline computing networks for achieving better detection performance. These schemes are given as follows:

- EEGNet. Samples from the single group are directly used as training data for EEGNet network training, and the testing data in the target domain are classified without domain adaption. For strong, medium, weak, and combined groups, the EEGNet network is subdivided into P3-sEEGNet, P3-mEEGNet, P3-wEEGNet, and P3-cEEGNet with the P3 map-clustering method for source domain selection.
- SDA. This is a DA method with a single source domain. Source samples from the single group are used as one source domain for domain alignment with the target samples. For strong, medium, weak, and combined groups, single domain adaption schemes are subdivided into P3-sSDA, P3-mSDA, P3-wSDA, and P3-cSDA with the P3 map-clustering method for source domain selection.
- MSDA. This is a DA method with multiple source domains instead of a single source domain. Individuals from the strong group are evenly divided into several source domains for domain alignment with the target samples. Here, sub-classifiers are trained for each source domain. The prediction results from the sub-classifiers are integrated to achieve the final classification. The MSDA scheme can be denoted as P3-MSDA when individuals with a strong P3 map are selected as source domains.

### Source Domain Selection

**Figure 5:**
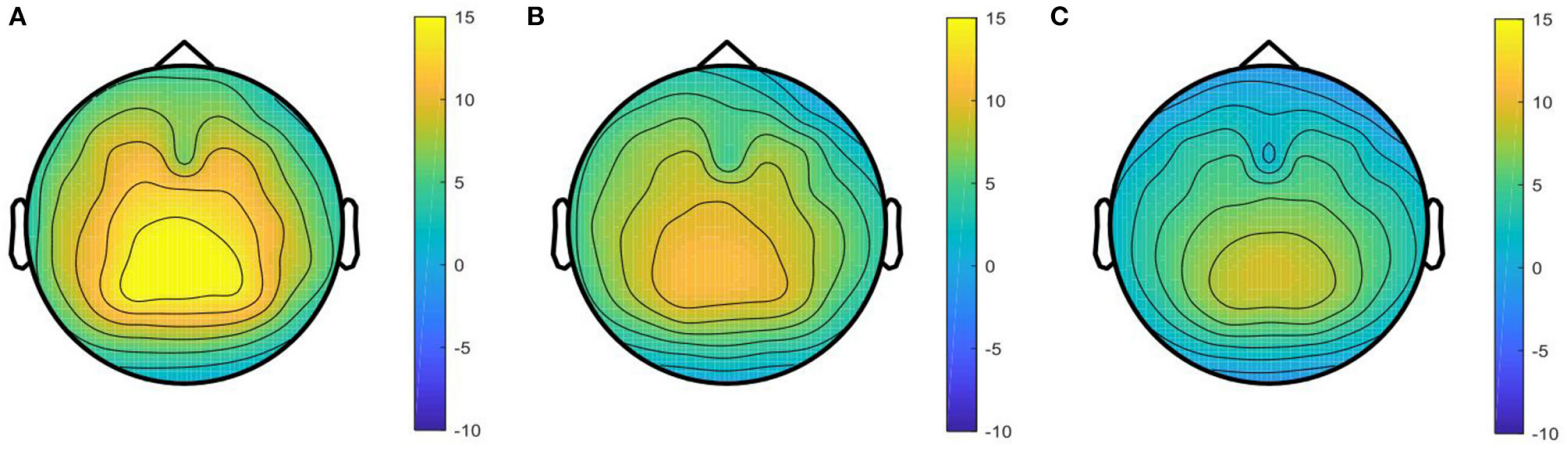
Averaged P3 map of the three groups. **(A)** Strong P3 map in, **(B)** medium P3 map in, and **(C)** weak P3 map in are from the strong P3 group, medium P3 group, and weak P3 group, respectively.

Based on theP3 map-clustering method, all individuals can be divided into three groups. The averaged P3 maps for each group are shown in Figure 5, including a strong P3 map group, **g**_1_ = {sub2, sub5, sub8, sub9, sub10, sub11, sub13, sub15, sub22, sub28}, medium P3 map group, **g**_2_ = {sub1, sub7, sub12, sub17, sub18, sub19, sub20, sub25, sub26}, and weak P3 map group, **g**_3_ = {sub3, sub4, sub6, sub14, sub16, sub21, sub23, sub24, sub27, sub29, sub30, sub31, sub32, sub33, sub34}.

### Detection Performance

**Figure 6:**
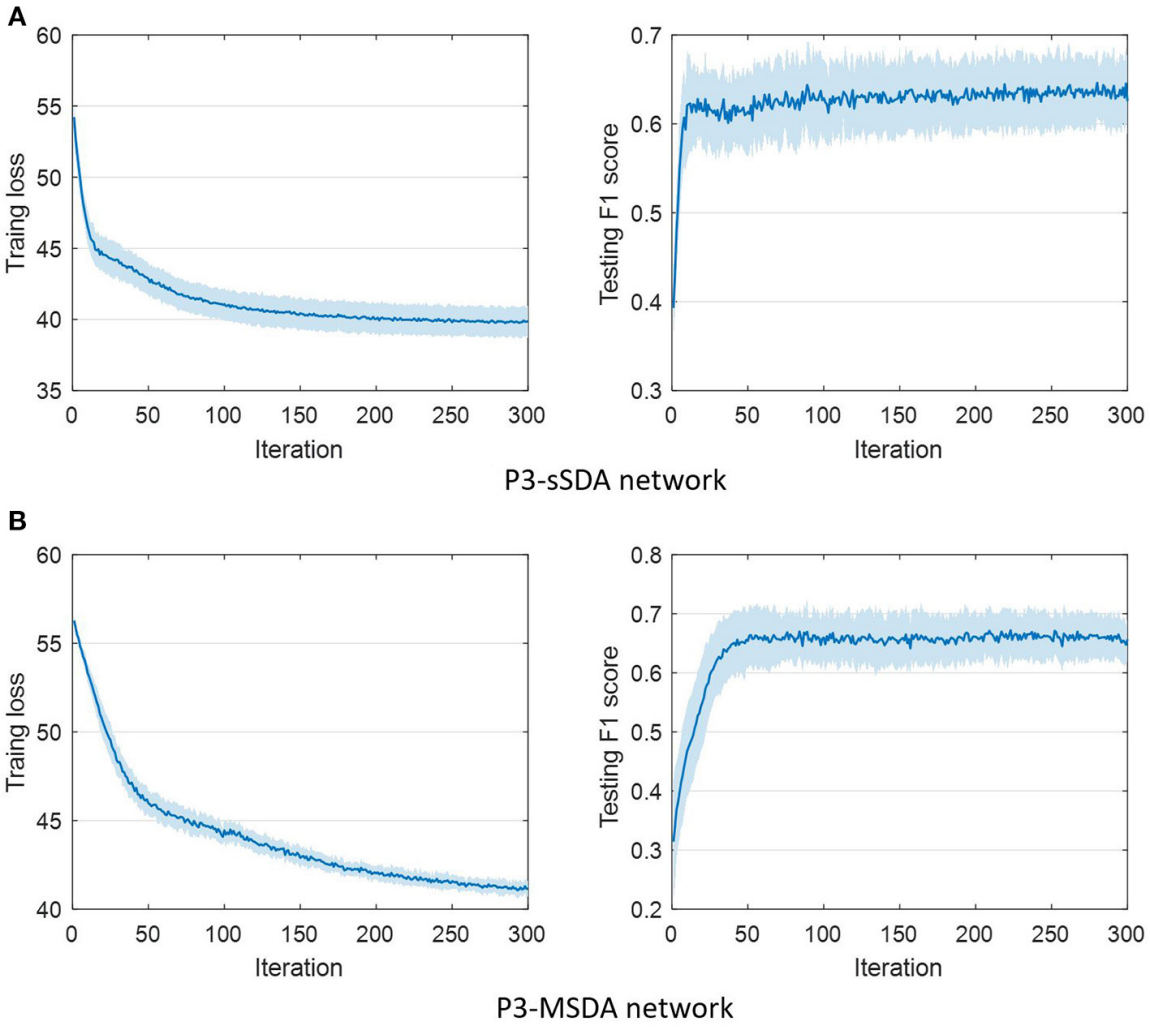
Convergence of training loss and detection performance in **(A)** P3-sSDA network and **(B)** P3-MSDA network.

In this study, F1 score is used for performance comparison because of imbalanced samples for the dynamic visual target detection task. The best detection performances of all schemes under different training individual numbers are summarized. They includeF1 score, classification accuracy, hit rate, false alarm rate, and significance level, as shown in Table 3, where the significance level calculated by ANOVA analysis evaluates the difference between P3-MSDA and others. During the experiment, the labeled individuals are selected from the corresponding groups as the training set or source domain. To ensure the randomness of individual selection, three individuals are respectively selected from the remaining individuals in the strong response group, medium response group, and weak response group as the target domain. The averaged results of three individuals represent the current detection ability. The experiment was randomly conducted 20 times, and their average value was considered the detection results. Among these schemes, the P3-MSDA network achieves the highest F1 score and classification accuracy, and performs significantly better than EEGNet and SDA. Although there is no significance between the P3-cSDA and P3-MSDA networks, the P3-MSDA network can perform better with fewer labeled individuals. For SDA schemes, the P3-cSDA network performs slightly better than the P3-sSDA network, while the P3-sSDA network can also perform well with fewer source domain individuals. The F1 scores of domain adaptation schemes are higher than those of EEGNet schemes. The optimal training individual number varies from 5 to 7 for this experiment. Correspondingly, the convergence of training loss and detection performance (F1 score) for the P3-sSDA network and the P3-MSDA network is analyzed in Figure 6. The training loss and F1 score can be gradually stable with 100 iterations and 20 iterations for the P3-sSDA network. Relatively, the P3-MSDA network can converge later than the P3-sSDA network. The training loss and F1 score can be gradually stable with 250 iterations and 50 iterations for the P3-MSDA network.

**Table 3:** Best detection performances of all schemes.

**Model**	**F1 score**	**Classification accuracy**	**Hit rate**	**False alarm rate**	**Labeled individuals**			***P* value**
					**Strong group**	**Medium group**	**Weak group**	
P3-sEEGNet	0.56	0.83	0.61	**0.12**	5	-	-	**
P3-mEEGNet	0.53	0.78	0.69	0.20	-	7	-	**
P3-wEEGNet	0.54	0.77	0.71	0.21	-	-	6	**
P3-cEEGNet	0.61	0.82	0.78	0.18	6	6	6	**
P3-sSDA	0.63	0.82	0.85	0.19	5	-	-	*
P3-mSDA	0.60	0.79	0.85	0.22	**-**	5	-	**
P3-wSDA	0.59	0.78	**0.86**	0.23	**-**	-	6	**
P3-cSDA	0.64	0.82	0.85	0.19	5	5	5	-
**P3-MSDA**	**0.66**	**0.84**	0.80	0.15	1,1,1,1,1	-	-	-

(**: p < 0.01, *: p < 0.05, -: p > 0.05).

*The bold values means the best value in each column*.

## Discussion

### Evaluation of Different Source Domain Selectors

Source domain selection is very crucial for the DA network. To verify the validity of the proposed P3 map-clustering method, three other criteria, namely, deviant ERP energy, energy ratio of deviation and standard ERP, and signal-to-noise ratio (SNR), are considered for clustering. The criteria of the P3 map and deviant ERP energy are proposed from a deviant sample perspective. However, the criteria of energy ratio and SNR are proposed based on the difference between deviant and standard samples. For each criterion, all individuals are clustered into three groups: strong, medium, and weak groups, according to the amplitude value.

- Deviant ERP energy: Since the negative wave at about 700 ms is as strong as the P3 component in the deviant ERP of video target detection, the entire deviant ERP response should be considered to balance more ERP components. Thus, the deviant ERP energy from the entire 1,000 ms with all channels is adopted as the individual feature for individual clustering.
- Energy ratio: The essence of EEG-based target detection is to distinguish between deviant and standard samples. Thus, the difference between deviant and standard samples should be highlighted for classification. Here, the energy ratio of deviation ERP and standard ERP can be viewed as a criterion for individual clustering. The higher the ratio, the greater the difference. The greatest difference group is the strong group.
- SNR: In addition to energy ratio, SNR can also reflect the overall signal quality. The higher SNR means better signals and performance. The actual collected signals consist of brain response and noise signals. Here, the brain response signals are denoted by the averaged ERP, and the noise signals are the difference between the collected signals and averaged ERP. The energy ratio of brain response and noise signals denotes the signal SNR. The greatest SNR group is the strong group.

Here, the EEGNet and SDA networks are used for evaluating the different clustering criteria. Samples from five labeled individuals are combined as source domain, and these individuals are from the same group. For each testing, one of the remaining individuals in each group is selected to form the target domain, and three target individuals from the strong, medium, and weak groups are considered. Finally, detection performance is obtained from the average value of 20-time random validation. The F1 score of detection performance with different clustering criteria is shown in Table 4, where performances on different source domains are analyzed. The highest F1 score is shown in bold for each case. For the same target individual, the detection performances on different source domains (strong, medium, and weak groups) are compared where a higher F1 score denotes the superiority of individuals as source domains. The results showed that the best performances for the EEGNet and SDA networks are produced from the strong source domain selected by the P3 map-clustering criterion. For the P3 map-clustering criterion, individuals from the strong group can act as the best source domain, and the SDA network with a strong P3 map achieves the best performance. Furthermore, the significance level between P3 map criteria with SDA scheme and other criteria is calculated by ANOVA analysis. Results indicate that the proposed P3 map-clustering method with SDA scheme can significantly improve the detection performance. These suggest that the proposed P3 map-clustering criterion, which can select active individuals as source domain to achieve better performance for the EEGNet and SDA networks, outperforms the three other criteria. Besides, the SDA network performs better than the EEGNet network with a **0.0**8 higher F1 score. Compared with the three other criteria, the P3 map criterion can contain the distribution and strength information of brain activity, which might explain why the P3 map criterion achieves the best performance. The response difference among brain regions from the strong P3 map group was maximized for training better classifiers.

**Table 4:** F1 scores of detection performance with different clustering criteria.

**Clustering criteria**	**Schemes**	**Performances on different source domains** **(strong, medium, weak)**	**Average**	***P* value**
P3 map	EEGNet	**0.56**, 0.51, 0.52	0.53	**
	SDA	**0.63**, 0.60, 0.58	**0.61**	**-**
Energy of deviant ERP	EEGNet	0.54, **0.54**, 0.51	0.53	**
	SDA	0.52, 0.52, **0.53**	0.52	**
Energy ratio	EEGNet	0.51, **0.54**, 0.51	0.52	**
	SDA	0.51, 0.52, **0.54**	0.53	**
SNR	EEGNet	**0.52**, 0.52, 0.51	0.52	**
	SDA	0.53, **0.54**, 0.51	0.53	**

(**: p < 0.01, -: p > 0.05).

*The bold values means the best value in each row*.

Furthermore, we test the effect of P3 map-clustering method on the target domain, as shown in Table 5. Here, we calculate the F1 scores when source domain and target domain are paired with different P3 intensity, using five source domain individuals. Results indicate that data from strong activation can perform best for both source domain and target domain. If the source data come from strong activation, the F1-score will get decreased with the target data changing from strong activation to low activation.

**Table 5:** Effects of P3 map-clustering method on the target domain.

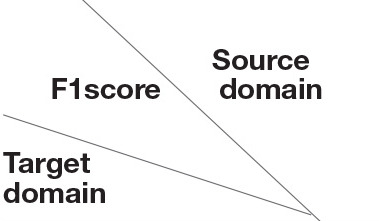	**Strong**	**Medium**	**Weak**
Strong	**0.68**	0.65	0.64
Medium	0.62	0.58	0.56
Weak	0.59	0.57	0.56

*The bold values means the best value in the whole table*.

### Effect of the Number of Labeled Individuals for Training

**Figure 7:**
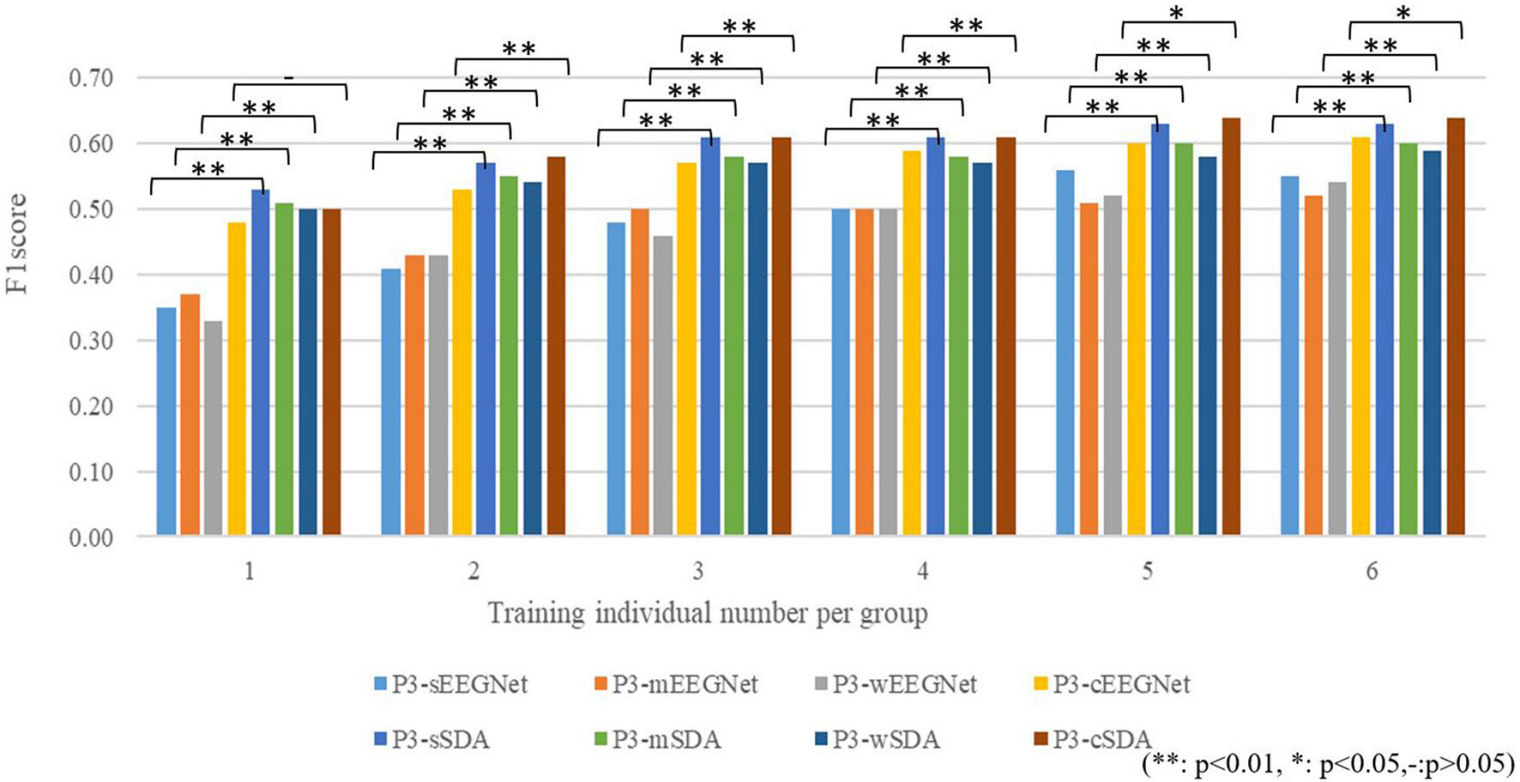
Effects of the number of labeled individuals for training.

Since the P3 map-based clustering criterion performs well for source domain selection, we compare the performance among the P3-sEEGNet, P3-mEEGNet, P3-wEEGNet, P3-cEEGNet, P3-sSDA, P3-mSDA, P3-wSDA, and P3-cSDA schemes, and explore the effect of the number of labeled individuals. The averaged F1 scores on the EEGNet network (P3-sEEGNet, P3-mEEGNet, P3-wEEGNet, and P3-cEEGNet) and the SDA network (P3-sSDA, P3-mSDA, P3-wSDA, and P3-cSDA) are respectively shown in Figure 7, where P3-cEEGNet and P3-cSDA combine individuals from three groups with three times individuals as the training set or source domain. The detection performance is given by the average value of 20-time random validation. The significance level of performance difference between EEGNet and SDA is calculated by ANOVA analysis. The results showed that the SDA network can significantly improve the F1 score, relative to the EEGNet network, and the performance can be improved with increasing labeled individuals. More labeled individuals do not always mean better performance. The best performance can be achieved with about five or six labeled individuals. For the EEGNet network, P3-cEEGNet achieves the best performance, especially for more training individuals. However, P3-sEEGNet performs better than P3-mEEGNet and P3-wEEGNet. For the SDA network, the P3-sSDA network can outperform P3-mSDA and P3-wSDA, and achieves an approximate detection effect with P3-cSDA. These findings further highlight the importance of individuals from the strong P3 group as the source domain and illustrate that more samples from the medium and weak groups cannot be beneficial to the performance of the SDA network. Thus, the P3-sSDA scheme with five to six training individuals from the strong P3 group is good as the single source domain for EEG-based dynamic visual target detection.

### Effect of the Number of Source Domains for the P3-MSDA Network

**Figure 8:**
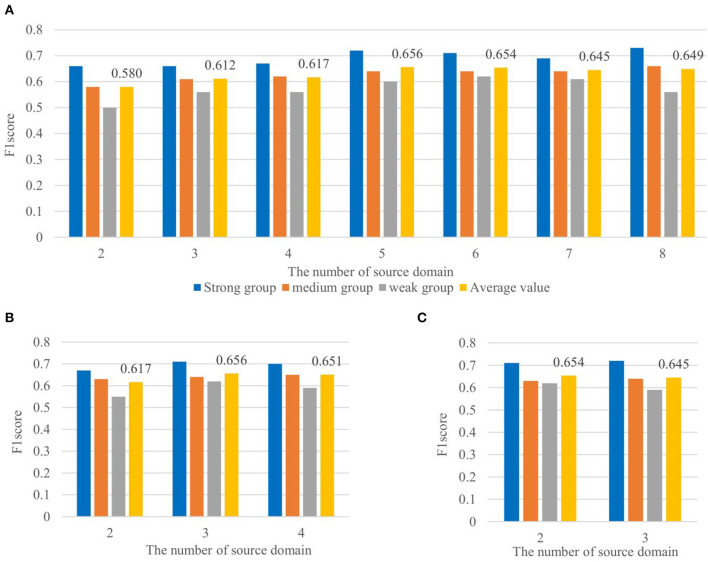
Effects of the number of source domains for the P3-MSDA network. The detection performances of target domain individuals from the strong, medium, and weak groups, and their average value are included. It shows the effect of the number of source domain with each source domain consisting of **(A)** one individual in, **(B)** two individuals in, and **(C)** three individuals in.

Based on the excellent performance of the P3-sSDA network, we developed a P3-MSDA network, where source domain individuals are selected from the strong P3 map group. Here, P3-MSDA can assume a single individual as one source domain or evenly divide all individuals into several source domains. The F1 scores of P3-MSDA are presented in Figure 8 where performances from the number of source domains are compared. For each case, three target individuals from different groups are randomly selected, and the averaged F1 scores are compared. The F1 score of the target domain performance from the strong, medium, and weak groups, and their average value are presented. The results showed that the individual number, rather than the source domain number, has a greater impact on the F1 scores of the P3-MSDA network. The best performance of P3-MSDA can be achieved with 5–6 training individuals, and target individuals from the strong P3 map group achieve the best performance, which is consistent with that of the SDA network. Besides, P3-MSDA outperforms P3-sSDA.

## Conclusion

In this study, we developed an unsupervised multi-source domain adaptation (P3-MSDA) network for dynamic visual target detection, which is an individual generalized model with imbalanced samples. In the P3-MSDA network, a P3 map-clustering method was proposed for source domain selection. The results showed that individuals with a strong P3 map could perform best as source domains, suggesting the superiority of individuals with high-brain activation levels for dynamic visual target detection. Besides, a target sample selector was designed to guide the input of target samples for imbalanced data classification. Based on these, the proposed P3-MSDA could exhibit higher classification accuracy and F1 score than the EEGNet network without domain adaptation and SDA networks with a single source domain, achieving an individual generalized model for dynamic visual target detection.

## Data Availability Statement

The original contributions presented in the study are included in the article/supplementary material, further inquiries can be directed to the corresponding author.

## Ethics Statement

The studies involving human participants were reviewed and approved by Henan Provincial People's Hospital. The patients/participants provided their written informed consent to participate in this study.

## Author Contributions

XS was mainly responsible for research design, data analysis, and manuscript writing of this study. YZ was mainly responsible for data analysis and manuscript writing. LT was mainly responsible for research design and data analysis. JS was mainly responsible for data collection. GB was mainly responsible for data analysis. BY was mainly responsible for research design and document retrieval. All authors contributed to the article and approved the submitted version.

## Conflict of Interest

The authors declare that the research was conducted in the absence of any commercial or financial relationships that could be construed as a potential conflict of interest.

## Publisher's Note

All claims expressed in this article are solely those of the authors and do not necessarily represent those of their affiliated organizations, or those of the publisher, the editors and the reviewers. Any product that may be evaluated in this article, or claim that may be made by its manufacturer, is not guaranteed or endorsed by the publisher.
